# The need for a food allergy educator program for allied healthcare professionals in Canada

**DOI:** 10.1186/s13223-022-00701-2

**Published:** 2022-07-07

**Authors:** Jennifer L. P. Protudjer, Carina Venter, Marion Groetch, Tara Lynn Mary Frykas, Jasmin Lidington, Harold Kim

**Affiliations:** 1 Department of Food and Human Nutritional Sciences, University of Manitoba, Winnipeg, MB Canada; 2 Canadian Society of Allergy and Clinical Immunology, Orleans, ON Canada; 3 Department of Pediatrics and Child Health, University of Manitoba, 501G-715 McDermot Avenue, Winnipeg, MB R3E 3P4 Canada; 4 The Children’s Hospital Research Institute of Manitoba, 501G-715 McDermot Avenue, Winnipeg, MB R3E 3P4 Canada; 5 George and Fay Yee Centre for Healthcare Innovation, Winnipeg, MB Canada; 6 Centre for Allergy Research, Karolinska Institutet, Stockholm, Sweden; 7 Children’s Hospital Colorado, University of Colorado, Denver, CO USA; 8 Division of Pediatric Allergy & Immunology, Icahn School of Medicine at Mount Sinai, Jaffe Food Allergy Institute, New York, USA; 9 Faculty of Medicine, University of Western Ontario, London, ON Canada; 10 Department of Medicine, McMaster University, Hamilton, ON Canada

**Keywords:** Allied health, Food allergy, Medical education

## Abstract

Owing to a collaborative approach to patient care, and a paucity of allergists in Canada, there is a need to develop a food allergy educational program for allied health care professionals in Canada. Such programs already exist in the United States and Britain. Herein, we describe the outcomes of recent conference proceedings to inform the educational needs for such a program. As part of the 76th Annual Meeting of the Canadian Society of Allergy and Clinical Immunology (CSACI), held virtually due to the COVID-19 pandemic, we hosted a virtual workshop on the need for a food allergy educator program for Canadian allied health professionals. This workshop was co-developed with the CSACI and an industry partner, and featured allergy specialist dietitians. Attendance was open to all conference delegates, and to allied health professionals. As part of the registration process, registrants posed diverse food allergy-related questions, ranging from how to use an epinephrine autoinjector, to daily management and, how to cure food allergy. A national food allergy educator program will empower both allergy and non-allergy specialist healthcare professionals to appropriately counsel patients. This virtually-delivered program will begin to close a gap in healthcare access resulting from the geographic size of Canada, as it will enhance allied healthcare providers’ confidence to provide evidence-based food allergy care appropriately for those with food allergy.

**To the editor:**

 In Canada, there are fewer than 250 allergists [1] to help support the estimated 6% of Canadians who live with probable food allergy [2]. Food allergy requires strict avoidance of the allergen, constant possession of an epinephrine autoinjector [3] and has negative financial [4, 5] and psychosocial [6, 7] consequences. Many patients and their families require healthcare management from other types of providers, including mental health professionals and dietitians. Allied health professionals, including psychologists, speech language pathologists, registered dietitians, nurses (including nurse allergy educators), pharmacists and respiratory therapists, are key partners in optimizing patient care [8]. Although food allergy programs for allied health professionals exist in the United States [9] and in Europe [10], there is a noticeable absence of such training for allied health professionals in Canada. In fact, healthcare professionals, including psychologists [11] and dietitians [12] report having inadequate training to support the needs of those with food allergy. This raises substantial concerns, as research from our group points toward a lack of knowledge regarding food allergy, outside specialty settings. Dietitians have described how “*they were afraid, they did not know what to do, they did not even know how to communicate with [food allergic] families”* [12]. This knowledge gap is echoed by allergists who reported that “*[education on basic dietary care] should be more part of our mandatory learning”* [11].

These concerns exist despite the fact that rates of pediatric allergy are at an all-time high [2, 3], with some evidence that rates are continuing to increase [13], and new allergens continue to emerge [14, 15]. There is presently no food allergy educator program for allied healthcare professionals in Canada. This knowledge gap is further exacerbated by the limited food allergy curricula in post-secondary education dietetics and nursing. For example, the Canadian Dietetic Registration Examination Preparation Guide has a single mention of food allergy, which is coupled with intolerances, in terms of nutrition care [16]. The National Council Licensure Examination for Registered Nurses, which is administered to determine entry level ability for incoming nurses in Canada and the United States, includes questions on emergency management of food allergy reactions, including anaphylactic reactions [17]. Missing however, are questions on broader topics, including food allergy prevention. To address these knowledge gaps, our long-term vision is to create a food allergy educator program for allied healthcare professionals across Canada, which will be overseen by the Canadian Society of Allergy and Clinical Immunology (CSACI), the professional association of allergists and clinical immunologists in Canada. In Canada, healthcare is administered by the provinces, which contributes to significant variation in medical care, including food allergy care, across the country. A national educator program would lead to more consistency with regards to clinical management of food allergy. Herein, we describe the outcomes of recent conference proceedings to inform the educational needs for such a program.

As part of the 76th Annual Meeting of the CSACI, held virtually due to the COVID-19 pandemic, we hosted a virtual workshop on the need for a food allergy educator program for Canadian allied health professionals. This workshop was co-developed with the CSACI and Nutricia, and featured allergy specialist dietitians; C Venter PhD RD and M Groetch MS RDN, the co-creators of the US-based food allergy education program for healthcare professionals. Attendance at this workshop was open to all conference delegates. In addition, the workshop was open to allied health professionals at no cost, although advanced registration was required. In this latter category, 68 allied health professionals registered. As part of the registration process, we queried their top questions about food allergy, the food allergy education material to which they would like to have access, and confidence working with patients with food allergy. Top allergy questions were diverse, and ranged from how to use an epinephrine autoinjector, to daily management and, how to cure food allergy. Food allergy educational materials were similarly diverse, but notably included materials for both patients as well as allied healthcare professionals. Participant-reported confidence working with food allergy ran the continuum of the scale, from 1–10.

An estimated 6%—or 3 million Canadians—live with food allergy [2], an estimate which does not directly count families and communities. As such, the creation of a food allergy educator program for health professionals in Canada is highly practical in the short term, as it addresses an immediate knowledge gap among non-allergy specialist healthcare professionals, in parallel with the creation of evidence-informed resources for use in their own practices. Our long term vision is to embed our food allergy educator program into healthcare professional curricula. To this end, and to better reflect food allergy-related issues more broadly, there is a need to further address food allergy-related issues in Canada including access to allergy-friendly food and allergy care for Canadians in rural and remote regions of our country. As well, there is a need to expand the curriculum to include adults with food allergy.

In summary, a national food allergy educator program will empower both allergy and non-allergy specialist healthcare professionals to appropriately counsel patients. This virtually-delivered program will begin to close a gap in healthcare access resulting from the geographic size of Canada, as it will enhance allied healthcare providers’ confidence to provide evidence-based food allergy care appropriately for those with food allergy. Downstream benefits include increased confidence and more effective use of healthcare providers’ time. Long-term plans include certification for continuing medical education credits for those who successfully complete the program. Notably, such certified programs exist in Canada for certified respiratory educators [18] and diabetes educators [19]. The ultimate success of a national food allergy educator program will be to increase food allergy management amongst affected families, decrease the number of allergic reactions, and prevent accidental deaths. Such programs already exist in the United States [9] and in Europe [10]. Such a program is long overdue in Canada.

## Data Availability

A recording of the workshop is available upon request.

## Abbreviation

CSACI: Canadian Society of Allergy and Clinical Immunology
